# Using principal components analysis to explore competence and confidence in student nurses as users of information and communication technologies

**DOI:** 10.1002/nop2.19

**Published:** 2015-06-08

**Authors:** Fern Todhunter

**Affiliations:** ^1^ School of Health SciencesThe University of NottinghamQueen's Medical CentreRoom B48B Floor South BlockNottinghamNG7 2UHUK

**Keywords:** competence, computers, computing skills, confidence, information and communication technologies, principal components analysis

## Abstract

**Aim:**

To report on the relationship between competence and confidence in nursing students as users of information and communication technologies, using principal components analysis.

**Design:**

In nurse education, learning about and learning using information and communication technologies is well established. Nursing students are one of the undergraduate populations in higher education required to use these resources for academic work and practice learning. Previous studies showing mixed experiences influenced the choice of an exploratory study to find out about information and communication technologies competence and confidence. A 48‐item survey questionnaire was administered to a volunteer sample of first‐ and second‐year nursing students between July 2008–April 2009. The cohort (*N *=* *375) represented 18·75% of first‐ and second‐year undergraduates. A comparison between this work and subsequent studies reveal some similar ongoing issues and ways to address them.

**Methods:**

A principal components analysis (PCA) was carried out to determine the strength of the correlation between information and communication technologies competence and confidence. The aim was to show the presence of any underlying dimensions in the transformed data that would explain any variations in information and communication technologies competence and confidence. Cronbach's alpha values showed fair to good internal consistency.

**Results:**

The five component structure gave medium to high results and explained 44·7% of the variance in the original data. Confidence had a high representation. The findings emphasized the shift towards social learning approaches for information and communication technologies. Informal social collaboration found favour with nursing students. Learning through talking, watching and listening all play a crucial role in the development of computing skills.

## Introduction

In higher education and health care, nursing students are required to use information and communication technology (ICT) resources. Electronic or ‘e’ devices are based on computers and computing structures, but may be called something else, for example the connection between the components of a traditional fixed computer; the monitor, keyboard, mouse and hard drive have been superseded with mobile technologies such as smartphones and tablets (Agosto & Abbas 2011, Dede 2011, Jung 2013). Mobile and fixed technologies are supporting the digitalization of paper records and virtual engagement in education and healthcare practice (Department of Health (DH) 2007a, 2007b, 2011, Quality Assurance Agency for Higher Education 2010, Belk 2013, Todhunter *et al*. 2013). All of which require a joint approach between ICT, the context for e‐learning and undergraduate pedagogy (Laurillard 2012). Nursing students need to be competent and confident to work with technologies that are constantly being updated.

In response, the Nursing and Midwifery Council's (NMC) interpretation of undergraduate fluency with technology has seen a shift from procedural rules and order, ‘Can use a variety of technological applications to record, store, retrieve and utilize information, while being alert to considerations of confidentiality and security.’ (NMC 2002, p. 18) to: ‘Interpret and utilise data and technology, taking account of legal, ethical and safety considerations in the delivery and enhancement of care.’ (NMC 2007, p. 8). The pedagogical recommendations are for experimental approaches and experiential learning with this interface, driven by the learner and not the technology (Quality Assurance Agency for Higher Education 2011, Laurillard 2012).

These outcomes show culminating technical ability and ICT literacy as essential for engagement with nursing theory and practice. Previous studies reveal variability in how nursing students deal with this environment. This body of work and the theoretical properties of competence and confidence shaped the study design. The findings showed that students value social interaction when learning about and learning using ICT.

### Background

The societal level of ICT engagement shows that it is difficult not to have had some kind of interaction with this environment. In comparison to other groups, healthcare learners are late recipients of teaching and learning for ICT, computers and computing. Established and more recent literature shows that nursing students have been caught up in the aftermath of an underprepared workforce (Willmer 2005, Bond & Procter 2009, Bond 2010, Sheikh *et al*. 2011, Cheng 2012). Collaborations between industry and higher education for workforce ICT preparation commenced circa 1970 with proactive global investment into workforce learning and development (Postle 1993, David & Foray 1994, Dummort & Dryden 1997, Schoenecker & Cooper 1998, Lamb *et al*. 2002, Royal Society 2012, World Bank 2013).

Similar investment in healthcare education appeared later. The bracketed citations reflect the serious attention given to addressing changes in work practice (DH 1997, 1998, 2002, 2005, 2007a, 2007b, 2011, 2012). Kennedy *et al*. (2010) showed that some long‐term computer users still struggle to learn with ICT and learn using ICT even at basic level. At societal level by the first quarter of 2012, 8·12 million (16·1%) adults in the UK (adults identified as 16+ years) had never used the Internet (Office of National Statistics (ONS) 2012). More recently, 32% of households cited lack of skills as a reason for no Internet access (ONS 2014). While product development advances, gaps in knowledge and skills between expert and basic users exist. Sections of the UK population continue to be at a disadvantage through lack of access and opportunity. This is a reflection of ongoing digital inequity (Litt 2013, Courtois & Verdegem 2014, Lüders & Brandtzaeg 2014).

Two studies reporting on nursing students’ computing prowess showed a preference for instructivist approaches, under‐confidence and reluctance to work independently (Koch *et al*. 2010, Moule *et al*. 2010). In contrast, a range of established and recent studies identify how nursing students are competent and confident working effectively with a range of computer‐based products (Ammenwerth *et al*. 2004, Oroviogoicoechea *et al*. 2006, Berglund *et al*. 2007, Garrat & Klein 2008, Kuosmanen *et al*. 2010, Windle *et al*. 2011). Studies with a specific focus on learning about the electronic healthcare record cite the influence of a supportive environment, developing a positive attitude and opportunities for training (Ballie *et al*. 2013, Kowitlawakul *et al*. 2015).

Given the ambitions of health care and the Department of Health's aim for a paperless environment by 2018 (Gov.uk, 2013), it essential that nursing students acquire computing competence and confidence through learning using and learning about ICT. Learning using ICT is working with computer‐based products used to store, manage and retrieve information for the development of professional knowledge and skills. It is important that students can use ICT to learn through it. This is distinguishable from learning about ICT as the merits of different artefacts to support participation, understanding and application.

Rapid changes and diffusion in product innovations have brought about new language and descriptions for computers and computing. Descriptions do not always include the noun ‘computer’ or the verb ‘computing’ in titles, acronyms or promotional material. Products still use computer‐origin interactive and inter‐connective components (Hoffman 2010). Indeed computer use and computing ability are synonymous with job and career opportunities (Lagesen 2008, Lemieux *et al*. 2009, Clark 2011, Lee 2013). Skills are organized as essential and/or desirable as a basis for recruitment. ‘Computing will be one of the fastest job growing markets through 2018.’ (Hoffman 2010, p. 19).

For this study, the computer was any device that houses software, connections, keyboard and monitor structures, irrespective of age, size and appearance, mobile or fixed, wireless or otherwise, that nursing students use for their studies. For example, computer‐based resources used to access ICT functions such as the Internet. Collectively, these artefacts are distinguishable from the computing skills that nursing students need to develop. Computing skills are the technical capital acquired for current studies and future professional practice. Examples are academic coursework and using patient‐related ICT resources in the clinical area such as the electronic healthcare record.

### Competence

The antecedents and outcomes for competence have been extensively studied. This discussion details some of the pragmatic self‐assessment qualities of competence that students work towards as: capability (Savolainen 2002, Mulder 2007, Lum 2009, Lozano *et al*. 2012), performance (Rummler & Brache 1995, Gilbert 1996), expertise (Dreyfus & Dreyfus 1986, Swanson 1994, Benner 2000) and being competent when presented with an unfamiliar task (Ammenwerth *et al*. 2004, Oroviogoicoechea *et al*. 2006, Berglund *et al*. 2007, Garrat & Klein 2008, Kuosmanen *et al*. 2010, Windle *et al*. 2011). These contexts represent the e‐learning competencies for classroom and patient‐related activities.

### Confidence

The vocabulary for describing confidence in learners as computer users focused on two areas. Confidence as self‐belief is personal in nature and associated with inner feelings such as having courage, feeling positive or negative (Koriat *et al*. 1980, Bandura 1986, Bowman 1999, Berman 2006). These feelings influence the response to a situation. Item‐specific confidence describes the interaction between two factors that influence confidence. These are what is believed to be correct and how confidently an individual displays their belief as behaviour in specific situations (Gigerenzer *et al*. 1991, Kruger & Dunning 1999, Butterfield & Metcalfe 2001, Whitehouse *et al*. 2002).

The combination of cognitive and social processes reflects the delicate balance between being able to cope or otherwise with ICT and computing activity. As such the observed behaviours can show a positive or a negative relationship between competence and confidence. To support the development of computing competence and confidence, in the classroom, students are given opportunities to participate in learning about and learning using ICT. In practice, entry level to patient records under Mentor supervision gives insight into postregistration accountability and responsibility with electronic health systems.

## The study

### Aim

The aim of this study was to find out if there was a relationship between computing competence and confidence; with a sub‐question of in what types of tasks and circumstances are these observable?

### Methodology

This study is an exploratory principal components analysis of self‐ reported data.

### Participants

Data were collected from first‐ and second‐year nursing students between July 2008–April 2009. A total of 382 students volunteered to participate in the research, representing 18·75% of the 2000 first‐ and second‐year students across the School of Nursing. Exactly, 375 students returned the signed consent form giving a 98% response rate from the initial volunteer group of students. Table 1 summarizes the characteristics of the cohort.

**Table 1:** Cohort characteristics

	*n*	%
Gender		
Female	322	89·4
Male	36	10
Missing	2	0·6
Age		
17‐20	96	26·48
21‐30	132	35·58
31‐40	104	27·5
41‐50	38	8·89
51‐60	4	1·29
Missing	1	0·26
Year of study		
Year 1	224	62·2
Year 2	128	35·6
Missing	8	2·2
Do you have access to a computer at home?		
Yes	357	95·2
No	3	0·8
Missing	15	4·0
What is your main reason for using a computer at home?		
Study	208	57·8
Leisure	142	39·4
N/A no computer at home	3	0·8
Missing	7	1·9
Please identify if your main previous activity involved the use of a computer		
Post‐16 years study	175	46·67
Paid work	96	25·60
Main previous activity did not involve the use of a computer	101	26·93
Missing	3	0·8
Have you undertaken previous formal study in computing knowledge and skills?		
Vocational	115	30·67
Academic	96	25·60
No formal study	162	43·20
Missing	2	0·53
Do you use a computer in the School of Nursing		
Yes	338	90·13
No	17	4·53
Missing	20	5·34

### Instrument

#### Survey development

A postal survey by questionnaire was preferable to survey by e‐mail attachment on the basis of bias towards respondents who were already interested in computers and ICT. The self‐assessment competence and confidence items were based on the cited theoretical frameworks and previous studies. Levett‐Jones *et al*.'s questionnaire measured competence and confidence in the use of a range of ICT applications including e‐mail and word processing (Levett‐Jones *et al*. 2009). Brettle and Raynor (2012) developed a questionnaire to assess online literature searching skills. Scores were assigned to activities such as truncation and being able to use Boolean operators. Moule *et al*. (2010) carried out a two‐phase study that included survey by questionnaire to explore nursing students’ experiences of e‐learning in higher education. Fetter (2009) measu‐red nursing students’ abilities and access to practice information systems. Bogossian and Kellett (2010) explored barriers to e‐portfolios and feeling comfortable when working with computers. Hoffman *et al*. (2011) asked nursing students to rate technical aspects of computing activities and their ability to access technical help.

#### Scale

The choice of scale and its adjectives required careful consideration. Brettle and Raynor (2012) asked their participants to rate their confidence from 1 (=confident)‐5 (=no confidence) in the use of several personal computer applications. Ragneskog and Gerdner (2006) used a five‐point scale from strongly agree to strongly disagree, with uncertain as the mid‐point in a non‐randomized convenience sample. Bogossian and Kellett (2010) used a four‐point scale with prefix adjectives to assess feeling comfortable when working with computers. Nursing students were asked to select from: extremely comfortable, very comfortable, somewhat comfortable and not comfortable. In phase 1 of her data collection, Bond (2009) asked nursing students to rate their skills to computing tasks as: excellent, good, basic or not used. Neutral is an indeterminate adjective and therein carries the risk of limitations in data generation and analysis (Johnston *et al*. 2003, Yaghmaie 2007). For this study, a four‐point adject‐ival ordinal scale was used to measure the responses (Strongly Disagree = 1, Disagree = 2, Agree = 3, Strongly Agree = 4).

### Validity and reliability

Having created the survey instrument, the next task was to determine its validity and reliability. Validity and reliability are intertwined because until a measure is reliable, it cannot be said to be valid. The purpose is to unearth any errors linked to the measurement.

#### Validity

Validity is the extent to which an instrument measures what it has been designed to measure, in that it will stand the test of time for subsequent research (Field 2009). These characteristics were assessed using face validity and construct validity. Face validity is an important practicality. It is used to detect anomalies associated with extraneous statements, logical language, syntax and any problems with random error in the questionnaire. This required the involvement of individuals as willing volunteers to assess sentence structure and implied meaning. Face validity was assessed by an independent group of nursing students and led to the removal of extraneous questionnaire statements and anomalies in logical language and syntax. Construct validity is the assessment of the nature of the variables and their relevance to the study. An expert colleague assessed the construct validity for each item. The final instrument was organized as eight categorical and 40 interval items, divided into 20 statements each for competence and confidence.

#### Reliability

**Figure 1:**
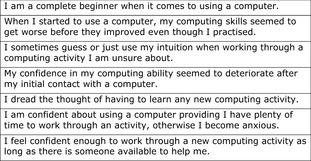
Reverse‐coded items for Cronbach's alpha.

Any inconsistencies in a set of questions will give unstable results (Tabachnick & Fidell 2014). Using Cronbach's alpha coefficient, the permutations tested for internal consistency were: positively worded scale items* *=* *0·87, items with reverse codes applied to negatively worded statements* *=* *0·78 (Figure 1), competence items with reverse codes applied to negatively worded items* *=* *0·615 and confidence items with reverse codes applied to negatively worded items* *=* *0·62. Cronbach's alpha for the final instrument* *=* *0·78 overall.

### Ethical considerations

The most important aspects of any research activity are the duty of care towards, protection of and respect for the participants. Documents and protocols for research using healthy volunteers were submitted to and approved by the University's Faculty of Health Sciences’ Medical School Ethics Committee and the Head of School. The documentation identified that this research would not have any bearing on the students’ status. Volunteers were given information relating to the study and their rights as healthy volunteers for coercion, consent, anonymity and confidentiality. All data were stored securely in Microsoft Excel by a designated administrator. Information was maintained and backed up on the organization's hard drive. Authorization was given to approach the student population by e‐mail and hard copy poster advertisement. Direct access to groups of students was also permitted, but only in the presence of another colleague and at the conclusion of a teaching session.

### Data analysis

Principal components analysis (PCA) is a statistical data reduction technique and part of the factor analysis family of methods used to explore linear relationships among a group of variables. It shows the level and importance of the variables and is sensitive to variance. PCA accommodates reliability test and retest before and after the analysis. It would reveal any correlation between competence and confidence. Data were entered into SPSS (version 16) and a PCA was conducted on the 40 items, using a direct oblimin (oblique) rotation. The aim was to obtain a parsimonious solution by explaining the variation in the original data set using a few underlying components (Tabachnick & Fidell 2014). Using pairwise deletion for missing data, item‐to‐subject ratios met the recommendations for sample size. Reliability tests of Kaiser–Meyer–Olkin (KMO) measure of sampling adequacy and communality values justified the use of PCA. With >300 cases and to improve the distinction between components, items with loadings <0·298 were suppressed.

Initial examination of the correlations was to ensure that there were correlations >0·3. This influenced whether to proceed or not with PCA (Field 2009). To reject the null hypothesis, Bartlett's test of sphericity needed to be <0·05. The practical utility of Bartlett's test is questionable as it reports significance irrespective of the quality of the study (Field 2009). Checks for multicollinearity and singularity were unremarkable. Components were extracted using the criteria for eigenvalues = 0·8 and examination of the scree plot and Monte Carlo PCA for Parallel Analysis (Watkins 2006). Given the human behavioural relationship between competence and confidence, it was anticipated that an oblique rotation would produce a realistic solution. This gave two matrices, the pattern matrix of unique relationships (no overlap among components) between each component and each variable and the structure matrix of the correlation between components and variables.

The *t*‐test determines whether the averages of two groups are statistically different (Tabachnick & Fidell 2014). *t*‐tests conducted in the factor regression scores gave mean differences between first‐ and second‐year students in the sample using the components generated by the PCA. The categorical variable of ‘year of study’ was chosen because it was the only one influenced by the University. It was anticipated that there would be relationship between competence and confidence. Using one categorical independent variable (year of study) and one interval‐dependent variable (regression scores for the five components), independent samples *t*‐tests were conducted to see if there were any significant statistical differences in mean scores of groups of participants. The statistical analysis for effect size was Eta squared. A Cronbach's alpha was calculated for each component.

## Results

At 0·875, the Kaiser–Meyer–Olkin (KMO) measure of sampling adequacy was classed as great. Therefore, the sample size at *n* = 375 was highly satisfactory for PCA. Bartlett's test of sphericity χ^2^ (780)* *=* *5·389, *P* < 0·001. This was an indication that correlation between items was well defined for a PCA. Eigenvalues set at 0·8 gave 16 components. Inspection of the scree plot and the data generated in parallel analysis confirmed five components as significant. Components 1‐5 totalled 44·7% of the variance in the original data, while components 6‐16 totalled 28% of the variance. Of the 16 components, 10 broadly reflected competence and 6 broadly reflected confidence. Based on the magnitude of the associated eigenvalues, the visual evidence of the scree plot and the Monte Carlo Parallel Analysis, the first five components were retained and explored (See Table 2). Examination of the residual components did not reveal anything significant. With the exception of component 5, Cronbach’ alpha values were fair to good internal consistency. The first five components gave a mix of variables for competence and confidence, though confidence had a higher representation. The average of the communalities = 0·73. This was above the baseline parameter of 0·7 as recommended by Field (2009) and explained a significant amount of the original data. The direct oblimin rotation yielded an interpretable simple structure. Eta squared gave a small effect across all five components. There was no statistical difference in the mean scores between first‐ and second‐year students, across all five components.

**Table 2:** Structure matrix of components

Items	Communalities	Unrotated components	Components															
			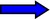 = one high value in the row								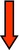 = several high and many low values in the columns							
			1	2	3	4	5	6	7	8	9	10	11	12	13	14	15	16
8	0·846	0·122	0·051	0·178	−0·073	−0·026	0·010	−0·016	0·130	−0·021	0·050	0·061	−0·012	0·007	−0·024	0·883	0·108	0·063
5	0·841	0·149	0·139	0·151	0·860	−0·055	0·246	0·045	0·012	−0·106	0·019	−0·006	−0·076	0·004	0·074	−0·098	0·002	−0·009
21	0·838	0·422	0·203	0·009	0·128	−0·022	−0·029	−0·226	0·128	0·085	−0·065	−0·119	−0·191	0·118	0·889	0·073	0·081	0·171
28	0·821	0·331	0·094	0·027	0·877	−0·144	0·253	−0·114	0·005	0·192	−0·110	−0·202	−0·136	0·155	0·076	0·087	0·122	0·094
29	0·807	−0·160	−0·077	0·087	−0·032	−0·102	−0·001	0·021	0·000	0·089	0·869	0·067	0·049	−0·071	−0·044	0·028	0·068	−0·040
33	0·799	0·001	−0·076	0·108	0·084	−0·873	0·135	−0·048	−0·011	−0·024	0·107	−0·007	−0·025	−0·074	−0·135	−0·006	0·034	0·100
17	0·795	0·135	0·139	0·067	0·026	0·048	−0·045	0·058	0·163	0·813	0·151	0·084	−0·053	−0·017	0·061	0·015	0·246	0·118
7	0·789	0·043	0·081	0·110	0·092	−0·856	0·039	−0·020	−0·009	−0·073	0·010	0·028	−0·023	−0·060	0·008	0·050	−0·093	−0·002
6	0·787	0·072	0·098	0·079	0·256	−0·090	0·854	0·104	−0·008	−0·115	0·043	0·062	−0·097	−0·025	−0·044	−0·042	−0·056	0·134
24	0·775	0·651	0·844	0·085	0·210	0·047	0·023	0·267	0·249	0·235	−0·125	−0·224	−0·264	0·316	0·209	0·162	0·029	0·207
27	0·765	0·268	0·014	−0·165	0·394	−0·090	0·683	−0·052	0·062	0·177	−0·086	−0·198	−0·012	0·296	0·034	0·226	0·232	0·094
35	0·765	0·517	0·316	0·138	0·153	−0·070	0·037	−0·218	0·237	−0·068	−0·087	−0·812	−0·252	0·203	0·163	0·052	0·044	0·205
12	0·764	−0·176	−0·134	0·105	−0·049	−0·481	0·146	−0·040	0·145	−0·219	0·113	0·301	−0·168	−0·518	−0·411	0·014	0·180	0·158
38	0·762	0·744	0·407	0·038	0·187	−0·270	0·195	−0·302	0·176	0·011	−0·305	−0·264	−0·561	0·640	0·278	0·172	−0·148	0·467
1	0·753	0·268	0·131	0·192	0·120	0·110	0·018	−0·061	0·192	0·261	0·035	−0·047	−0·132	0·109	0·132	0·182	0·816	0·115
37	0·747	0·770	0·503	0·028	0·197	0·031	−0·031	−0·408	0·207	0·229	−0·327	−0·364	−0·265	0·607	0·466	0·285	−0·070	0·429
26	0·745	−0·496	−0·146	0·149	−0·254	0·056	0·080	0·497	−0·080	−0·405	0·365	0·549	0·232	−0·161	−0·142	−0·216	0·191	−0·159
11	0·739	0·225	0·016	0·429	−0·002	−0·419	0·124	−0·303	0·332	0·171	−0·210	0·160	−0·398	−0·023	−0·142	0·214	−0·097	0·005
2	0·727	0·092	−0·094	0·672	0·008	−0·121	0·042	−0·034	0·117	0·100	−0·019	−061	0·061	0·092	0·148	0·113	0·517	−0·022
10	0·724	0·286	0·142	0·019	−0·076	−0·024	−0·074	−0·172	0·818	0·099	−0·029	0·018	−0·080	0·057	0·065	0·286	0·168	0·102
23	0·721	0·479	−0·392	0·078	−0·078	0·031	0·066	0·782	−0·115	0·018	0·061	0·184	0·130	−0·153	−0·136	−0·066	−0·051	−0·313
9	0·713	0·346	0·196	0·078	0·100	0·027	0·047	−0·051	0·806	0·087	−0·017	−0·200	−0·209	0·084	0·086	0·034	0·077	0·097
18	0·712	0·575	0·331	−0·008	0·239	−0·032	−0·323	−0·311	0·214	0·312	−0·051	−0·061	−0·616	0·329	0·226	0·294	0·061	0·228
3	0·709	0·106	0·503	0·810	0·197	−0·128	−0·018	0·028	0·042	−0·003	0·042	−0·054	−0·079	0·022	−0·012	0·128	0·088	0·003
25	0·706	−0·676	−0·673	0·127	−0·125	0·040	−0·071	0·407	−0·242	−0·145	0·386	0·214	0·165	−0·304	−0·274	−0·254	−0·147	−0·424
30	0·705	0·500	0·301	0·052	0·180	−0·297	−0·022	−0·228	0·377	0·112	−0·031	−0·157	−0·144	0·150	0·013	0·343	0·398	0·638
13	0·700	0·531	0·274	−0·044	0·239	−0·118	0·201	−0·120	0·189	−0·064	−0·128	−0·031	−0·507	0·568	−0·014	0·090	0·071	0·472
14	0·699	−0·599	−0·405	−0·008	−0·129	0·001	−0·018	0·410	−0·202	0·042	0·142	0·281	0·222	−0·724	−0·189	−0·208	−0·217	−0·090
15	0·697	−0·405	−0·139	0·037	−0·052	0·011	0·109	0·800	−0·133	−0·064	0·115	0·173	0·134	−0·228	−0·288	−0·161	0·009	−0·006
34	0·697	0·763	0·705	0·008	0·267	−0·067	0·086	−0·318	0·243	0·085	−0·188	−0·479	−0·426	0·333	0·326	0·238	0·105	0·324
39	0·694	0·519	0·261	0·041	0·052	−0·011	0·184	−0·194	0·033	0·194	−0·203	−0·258	−0·261	0·153	0·334	0·127	−0·005	0·739
20	0·694	0·695	0·380	0·130	0·159	−0·112	0·130	−0·376	0·348	−0·050	−0·204	−0·500	−0·636	0·231	0·312	0·138	0·104	0·280
31	0·694	0·652	0·347	0·000	0·214	0·019	0·060	−0·190	0·106	0·330	−0·516	−0·449	−0·538	0·371	0·277	0·130	0·080	0·231
32	0·658	0·752	0·558	−0·065	0·159	0·012	0·052	−0·372	0·163	0·236	−0·292	−0·431	−0·479	0·343	0·452	0·187	0·026	0·372
4	0·668	0·082	0·115	0·641	−0·002	−0·069	0·172	0·091	0·150	0·067	0·343	−0·051	−0·027	−0·151	−0·059	0·290	0·183	0·241
19	0·651	0·666	0·610	0·018	0·327	−0·044	0·080	−0·328	0·322	0·038	−0·063	−0·296	−0·521	0·156	0·246	0·355	0·060	0·221
40	0·650	0·689	0·485	−0·017	0·135	0·078	−0·065	−0·249	0·388	0·147	−0·185	−0·333	−0·185	0·522	0·353	0·315	0·047	0·490
36	0·645	0·604	0·343	0·069	0·181	0·082	0·034	−0·144	0·249	0·124	−0·153	−0·330	−0·719	0·248	0·339	0·126	−0·351	0·217
22	0·602	0·677	0·526	0·001	0·234	−0·234	−0·001	0·402	0·343	0·027	−0·113	−0·165	−0·308	0·379	0·336	0·415	0·065	0·353
16	0·598	0·598	0·316	0·044	0·220	0·042	0·083	−0·365	0·413	0·160	0·025	−0·099	−0·243	0·435	0·281	0·232	−0·003	0·522
			1	2	3	4	5	6	7	8	9	10	11	12	13	14	15	16

**Figure 2:**
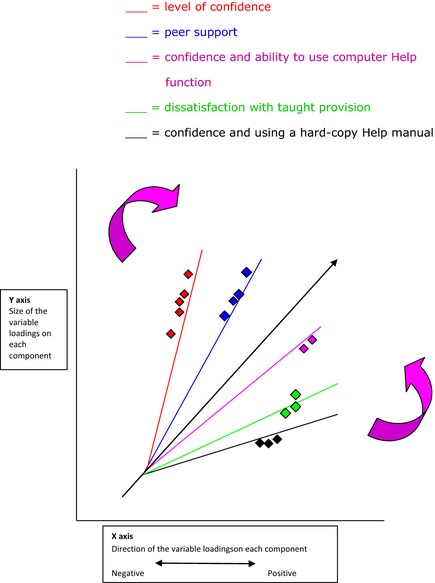
Geometric interpretation of first five components with oblique rotation.

Figure 2 is a cross‐section of a geometrical overview for the first five components. The loadings have been placed to approximately represent the figures in Table 2. Using an oblique rotation, Figure 2 shows the relationship between each component and its variables. The X‐axis shows the direction of the variable loadings (positive or negative) on each component. The Y‐axis reflects the size of the variable loadings on each component. Figure 2 shows clusters of several variables at midline and far out on each component. Higher loadings are the furthest away from the origin. The lowest loadings are the nearest to the point of origin. Both are indicative of reasonably and well‐defined components (Field 2009). As the diagram shows the first five components, the space has five axes and five dimensions, and each observed variable is positioned by five co‐ordinates. The clusters also show the closeness of the variables and the shared variance between them. The oblique rotation allowed the components to correlate. This showed where the variables correlated with one component through indirect correlation with another component.

Loadings in components 1, 4 and 5 (placed to the right) represent the negative correlations with the axis. The component 2 shows the high and medium positive cluster for positive peer support and the variables of listening, watching and talking. The component 3 shows a high positive cluster for nursing students using online utilities.

### Component 1: confidence

The results show opinions on working independently in the ICT environment. The negative medium‐sized loading for ‘I dread the thought of having to learn any new computing activity’ is comparable with the medium‐sized loading for ‘I am confident when asked to do a new computer exercise as long as there is someone available to help me.’ These results show the dominance of confidence over competence.

### Component 2: opinions about peer support and learning styles

The component 2 identifies that nursing students value and benefit from working in informal learning groups. Removal of Q19 ‘Using a computer in the University has assisted in the organization/management of my studies’ improved the Cronbach's alpha (=0·643). The loadings in the second component show the development of competence through listening and talking to and watching others using ICT. These variables reflect the links between technology and human interaction.

### Component 3: opinions about using computer ‘Help’ functions

The component 3 shows two equally high loadings for both competence and confidence when using the ‘Help’ features on a computer. This component reflects positive aspects for problem‐solving and feeling confident to use and respond to the Help function.

### Component 4: opinions about taught provision

The component 4 shows two high negative loadings competence and confidence and teaching. The combined loadings show that taught ICT provision had not influenced computing skills and knowledge. This outcome reflected significant dissatisfaction with teaching and learning resources at that time.

### Component 5: opinions about using hardcopy Help manuals

The two medium to high loadings reflect competence and confidence in following and applying written hard copy ICT instructions. The removal of Q26 ‘Sometimes I can complete certain computing activities without even having to think about them’ marginally improved the Cronbach's alpha = 0·554.

## Discussion

The goal was to find out if there was a relationship between computing competence and confidence and establish the types of tasks and circumstances where these phenomena could be observed. Several themes emerged from the components.

### Theme 1: nursing students working independently as computer users

The loadings on the first component show that students were confident in the ICT environment and concur‐red with previous findings (Ammenwerth *et al*. 2004, Oroviogoicoechea *et al*. 2006, Berglund *et al*. 2007, Garrat & Klein 2008, Kuosmanen *et al*. 2010, Windle *et al*. 2011). The tasks and circumstances where confidence could be observed were: nursing students working independently, tackling unfamiliar activities, being able to complete a computing task and feeling confident enough to ask for help when required. These findings support the underpinning theory for item‐specific confidence and responsive confidence as the courage to take action. More recent studies show that in spite of educational measures to support the cited tasks and circumstances, nursing students are still struggling to achieve ICT proficiency (Lee & Clarke 2015). In observing the ICT boundaries set between academic and work disciplines, Thorpe and Edmunds’ suggest creating practice learning to reflect the latter (Thorpe & Edmunds 2011). This shifts the focus from the ability of the individual student to work independently, to the responsibility of organizations to collaborate on integrating ICT. Pobocik (2014) recommends facilitating access to educational electronic healthcare documentation. Together these papers reflect the proactive creation of clinical ICT in a safe learning environment for the development of competence and confidence.

### Theme 2: peer support and interconnected personal learning experiences

The mutual sharing of time, ideas and support aligns with recommendations for peer groups and blended ICT learning arrangements (Quality Assurance Agency for Higher Education 2011). The mean scores showing no statistical significant difference between first‐ and second‐year students on the factor regression scores was unexpected. The cautious assumption that senior learners might be more competent and confident was refuted. These findings should be treated with caution as they offer limited understanding about different kinds of peer support related to ICT. Cheng and Chau (2014) highlight the significant differences depending on the activity and the subtle shifts from social to cognitive behaviours. For example, their findings for peer support and networked learning (working together online) reflect social interaction. In contrast, the creation of individual digital artefacts followed by showcasing this work to their peers, influences individual critical reasoning skills. Peer support is secondary to the learning. Both approaches enhance the learning experience.

### Theme 3: the usefulness of online and paper Help manuals

This theme represents the combined loadings for components 3 and 5 as nursing students’ opinions on resources other than human support. The observable tasks where competence and confidence can be seen are nursing students identifying that they can access and use different ICT support systems. Learning with and learning using ICT is significant both for study and professional practice (Brettle & Raynor 2012). Such experiences support familiarity with different products and skills (NMC 2007). This cannot be underestimated given the impact of ICT in patient care and the NMC requirement. These findings reflect ICT skill acquisition through following instruction and concur with Liu *et al*. (2013). Their results give a correlation between good information‐seeking skills and independent problem‐solving.

### Theme 4: the influence of taught provision on computing competence and confidence

The high and medium negative loadings on component 4 for taught provision show dissatisfaction with direct teaching. The observable tasks and circumstances for this correlation are complex as students engage in cyberspace with technologies beyond University provision and direct observation (Table 1). Mandatory outcomes (NMC 2007) require direct assessment of skill and knowledge development to determine level and kind of teaching. The increase in remote activity through ICT integration offers new opportunities for teaching pedagogy (Laurillard 2012). These findings suggest flexible opportunities for taught provision as indicated in component 1. ‘I am confident when asked to do a new computer exercise as long as there is someone available to help me.’ This concurs with two similar studies considering the circumstances where adults are likely to learn ICT successfully. Hughes *et al*. (2014) cited the benefits from a blend of: regular repeated practical sessions, PowerPoint instructions written in a simple, supportive dialogue, opportunities for discussion and access to the same facilitator. Their approach shifts the emphasis from the technology to the vulnerability of the learner. Lee *et al*'s exploratory factor analysis revealed the underlying value of developing learning in a direct human context before engaging students in a technological interface (Lee *et al*. 2014). With the forecasted increase in online learning, Meyer's report on what works and why, recommends further research into engagement and bespoke systems of student support (Meyer 2014).

### Limitations

The sample met the criteria for a principal components analysis. However, at >18% of the years 1 and 2 student nurse population, the findings should be treated with caution. The scale was designed to measure current competence and confidence levels and was unlikely to remain stable over a period of time. Principal components analysis summarizes the variance in the constructs of competence and confidence. It cannot claim to be definitively measuring these constructs. Albeit a method for simple identification and classification PCA has a reputation for being arbitrary. Several variables may provide similar information. Some which appear to be of significance provide no useful information. Arbitrary decisions on eigenvalue cut‐off points carry a risk of discarding substantive components. The choice of rotation was oblique. This decision was based on the literature and a possible relationship between competence and confidence. Examination of the R matrix and the components before rotation were indicative of the correlation. The oblique rotation and positioning of the clusters provided confirmation. Some of the discarded components close to zero may have produced a solution similar to that in an orthogonal rotation. The relationship between the individual questions and the components is empirical. The underpinning theory about which statements should load onto which components was selected from a broad interpretation. Another body of theory, different cohort or even this one at another time may give contrasting results. In spite of the choice of postal questionnaire, this study may have attracted only respondents skilled and interested in ICT.

## Conclusion

Survey by questionnaire and PCA gave a positive correlation between computing competence and confidence in nursing students as users of ICT and computer‐related resources. The observable features of competence emerged as being capable and working methodically with computers and employing computing skills for study activities. Confidence emerged as both item‐specific and responsive; the behavioural aspects for dealing with the familiar and unfamiliar. The results concurred with previous work showing nursing students working effectively and efficiently. Students identified favourably with learning through talking, listening and watching. The Faculty have now put into practice a combination of curricula and taught provision approaches in response to students’ teaching and learning needs. These include bespoke one‐to‐one teaching, the employment of expert learning technologists actively involved with pedagogy and student support, incremental challenges to support competence and the development of item‐specific and responsive confidence, small group learning activities, synchronous and asynchronous online Help communication and pictorial Help instructions. This study has shown the importance of assessing nursing students in the acquisition of computing competence and confidence in the ICT and computer environment. The increasing adoption of ICT and its related products in nurse education and health care will require an ongoing strategic and operational review of student support, professional learning outcomes and curricula activity.

## Conflict of interest

None.

## Author contributions

All authors have agreed on the final version and meet at least one of the following criteria [recommended by the ICMJE (http://www.icmje.org/recommendations/)]:

- substantial contributions to conception and design, acquisition of data, or analysis and interpretation of data;
- drafting the article or revising it critically for important intellectual content.
